# Metabolic profiles of 2-oxindole-3-acetyl-amino acid conjugates differ in various plant species

**DOI:** 10.3389/fpls.2023.1217421

**Published:** 2023-07-18

**Authors:** Pavel Hladík, Ivan Petřík, Asta Žukauskaitė, Ondřej Novák, Aleš Pěnčík

**Affiliations:** ^1^ Laboratory of Growth Regulators, Institute of Experimental Botany, The Czech Academy of Sciences & Faculty of Science, Palacký University, Olomouc, Czechia; ^2^ Department of Chemical Biology, Faculty of Science, Palacký University, Olomouc, Czechia

**Keywords:** auxin metabolism, auxin conjugates, HPLC-MS/MS, indole-3-acetic acid, 2-oxindole-3-acetic acid, catabolism, quantitative analysis

## Abstract

Auxins are a group of phytohormones that play a key role in plant growth and development, mainly presented by the major member of the family - indole-3-acetic acid (IAA). The levels of free IAA are regulated, in addition to *de novo* biosynthesis, by irreversible oxidative catabolism and reversible conjugation with sugars and amino acids. These conjugates, which serve as inactive storage forms of auxin and/or degradation intermediates, can also be oxidized to form 2-oxindole-3-acetyl-1-O-ß-d-glucose (oxIAA-glc) and oxIAA-amino acids (oxIAA-AAs). Until now, only oxIAA conjugates with aspartate and glutamate have been identified in plants. However, detailed information on the endogenous levels of these and other putative oxIAA-amino acid conjugates in various plant species and their spatial distribution is still not well understood but is finally getting more attention. Herein, we identified and characterized two novel naturally occurring auxin metabolites in plants, namely oxIAA-leucine (oxIAA-Leu) and oxIAA-phenylalanine (oxIAA-Phe). Subsequently, a new liquid chromatography–tandem mass spectrometry method was developed for the determination of a wide range of IAA metabolites. Using this methodology, the quantitative determination of IAA metabolites including newly characterized oxIAA conjugates in roots, shoots and cotyledons of four selected plant models - *Arabidopsis thaliana*, pea (*Pisum sativum* L.), wheat (*Triticum aestivum* L.) and maize (*Zea mays* L.) was performed to compare auxin metabolite profiles. The distribution of various groups of auxin metabolites differed notably among the studied species as well as their sections. For example, oxIAA-AA conjugates were the major metabolites found in pea, while oxIAA-glc dominated in Arabidopsis. We further compared IAA metabolite levels in plants harvested at different growth stages to monitor the dynamics of IAA metabolite profiles during early seedling development. In general, our results show a great diversity of auxin inactivation pathways among angiosperm plants. We believe that our findings will greatly contribute to a better understanding of IAA homeostasis.

## Introduction

The plant hormone auxin is involved in many growth and developmental processes. For the regular control of these processes, it is necessary to create local auxin gradients within cells and organs, which are mainly regulated by biosynthesis, polar transport, and metabolism. Although auxin metabolic pathways and endogenous levels of individual metabolites are well characterised in Arabidopsis, auxin biosynthesis and metabolism are diverse among the plant kingdom (Matsuda et al., 2005; Kai et al., 2007b; Sugawara et al., 2015; Brunoni et al., 2020; Brunoni et al., 2023). However, a full understanding of the biochemical processes in economically important crop plants is needed to better prepare for the ever-increasing demands. The Poaceae is the most agriculturally grown plant family. Maize, rice and wheat are the world’s most important source of food, with more than 600 million tons each being harvested annually (Shewry, 2009). Unfortunately, increasingly demanding growth conditions caused by climate change are reducing their yield. Another highly cultivated family is the Fabaceae, which are able to process nitrogen from the atmosphere through their symbiosis with nitrogen-fixing bacteria (Broughton et al., 2003). Pea, an important member of this family is used as a model plant since the beginning of the genetics research and was also used in the early auxin research to confirm its role in apical dominance (reviewed in Smýkal, 2014). Elucidation of auxin metabolic pathways in these species should lead to a better understanding of plant growth and adaptation to extreme conditions.

The most important auxin indol-3-acetic acid (IAA) can form biologically inactive metabolites via an amide bond with amino acids, peptides, and proteins or through an ester bond with glucose, inositol, and glucan (reviewed in Seidel et al., 2006; Normanly, 2010; Ludwig-Müller, 2011; Casanova-Sáez et al., 2021). In Arabidopsis, amide conjugates constitute 78% to 90% of the total IAA pool (Tam et al., 2000; Ljung et al., 2002), whereas in monocots IAA predominantly exists in the form of IAA esters (Ludwig-Müller, 2011). For example, in rice seeds, esters constitute 68% to 70% of the total IAA pool (Bandurski and Schulze, 1977). The conjugation of IAA with amino acids is mediated by a group of GRETCHEN HAGEN 3 (GH3) enzymes (Staswick et al., 2005). The most abundant IAA amino acid conjugates indole-3-acetyl-aspartic acid (IAA-Asp) and indole-3-acetyl-glutamic acid (IAA-Glu) have been determined in a number of plant species such as Arabidopsis (Östin et al., 1998; Novák et al., 2012), rice (Matsuda et al., 2005) or spruce (Brunoni et al., 2020). Indole-3-acetyl-alanine, indole-3-acetyl-leucine (IAA-Leu) (Kowalczyk and Sandberg, 2001) and indole-3-acetyl-tryptophan (Staswick, 2009) have been also detected in Arabidopsis. Furthermore, endogenous levels of IAA conjugates with glycine, phenylalanine (IAA-Phe) and valine were determined in seeds of *Helleborus niger* (Pěnčík et al., 2009). Besides conjugation, IAA may be inactivated by oxidation to 2-oxindole-3-acetic acid (oxIAA). Until recently, this mechanism was considered to be the main catabolic pathway of IAA (Östin et al., 1998; Woodward and Bartel, 2005; Pěnčík et al., 2013). Oxidized IAA can be further glucosylated to 2-oxindole-3-acetate-glucosyl ester (oxIAA-glc) (Tanaka et al., 2014).

**Figure 1:**
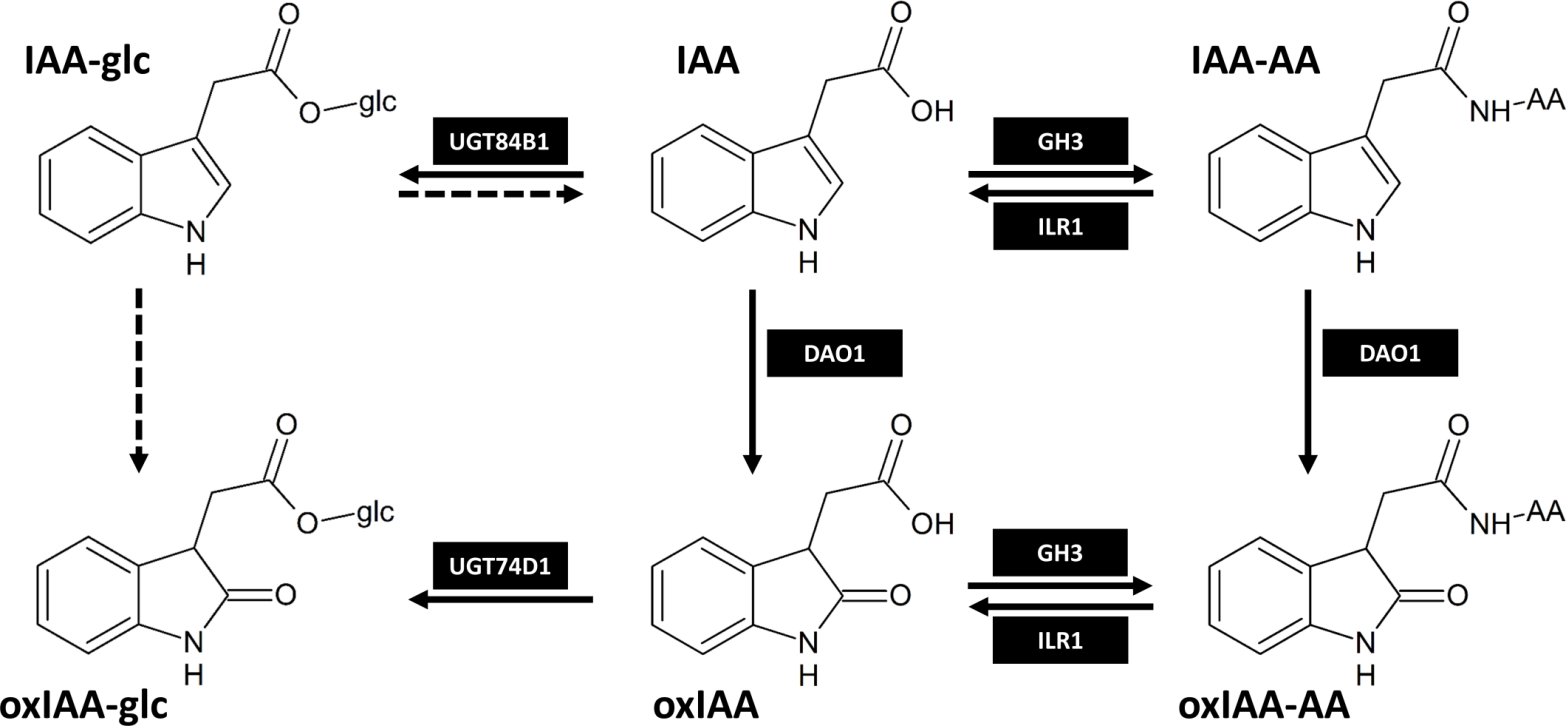
The main IAA inactivation pathways in Arabidopsis thaliana as proposed by Tanaka et al., 2014; Hayashi et al., 2021; Müller et al., 2021 and Brunoni et al., 2023. Dashed arrows represent metabolic steps that are still not fully described. IAA-AA and oxIAA-AA represent amide-linked conjugates with amino acids. The enzymes responsible for IAA metabolism shown in black boxes are as follows: DAO1, DIOXYGENASE FOR AUXIN OXIDATION 1; GH3, GRETCHEN HAGEN 3; UGT84B1/UGT74D1, UDP-glucosyltransferase 84B1/74D1; ILR1, IAA-Leu-Resistance 1.

There is also evidence of oxidation of IAA-Asp to 2-oxindole-3-aspartic acid (oxIAA-Asp). Application of exogenous IAA to branches of aspen (*Populus tremula* L.) led to an increase in IAA-Asp concentration and later formation of oxIAA-Asp (Plüss et al., 1989). Arabidopsis plants supplemented with exogenous IAA accumulated endogenous IAA-Asp, IAA-Glu, and oxIAA-Asp, while formation of oxIAA-Glu was not observed (Östin et al., 1998). When feeding Arabidopsis with deuterium-labeled IAA-Valine and IAA-Phe, the formation of oxIAA-Valine and oxIAA-Phe was confirmed, but no endogenous levels were detected (Kai et al., 2007a). Recent research revealed that DIOXYGENASE FOR AUXIN OXIDATION 1 (DAO1) is responsible for the conversion of IAA-Asp to oxIAA-Asp in Arabidopsis (Müller et al., 2021). oxIAA-Asp can be further converted to corresponding high molecular weight oxIAA-peptides (Riov and Bangerth, 1992) or 3-hydroxy-oxIAA (Tsurumi and Wada, 1986; Hayashi et al., 2021). So far, only oxIAA-Asp and oxIAA-Glu have been determined as endogenous metabolites in Arabidopsis (Hayashi et al., 2021), oxIAA-Asp in roots of rice (Isobe and Miyagawa, 2022), lychee leaves, flowers (Kim et al., 2021) and ovaries (Osako et al., 2022), while oxIAA-Glu was also detected in lychee leaves (Kim et al., 2021). In rice, oxIAA-Glu was not detected in the tissue, but it was present in hydroponic medium after stress treatment, thus suggesting its formation in rice (Isobe and Miyagawa, 2022). For a long time, the GH3-mediated conjugation and irreversible oxidation of IAA by DAO1 were believed to be two parallel pathways of auxin inactivation. Hayashi et al. (2021) showed that in Arabidopsis DAO1 preferably oxidizes IAA-amino acid conjugates IAA-Asp and IAA-Glu into oxIAA-Asp and oxIAA-Glu, respectively, which are subsequently hydrolysed by IAA-Leu-Resistance1/Arabidopsis ILR1-Like hydrolases (ILR1/ILL) to inactive oxIAA. Both mechanisms therefore contribute to the same auxin inactivation pathway (**Figure 1**). Recently, oxIAA conjugation mediated by GH3 enzymes has also been confirmed, however, its contribution to IAA homeostasis is species-dependent (Brunoni et al., 2023).

Auxins are present in plants in trace amounts, making their quantitative and qualitative analysis very challenging (Fu et al., 2011; Du et al., 2012; Porfírio et al., 2016). Many solvents are used for extraction, such as organic solvents or aqueous buffers (reviewed in Du et al., 2012). Recently, sample purification by solid-phase extraction (SPE) has become the most used method of auxin purification with the tendency to minimize the amount of solvents and analytes (Liu et al., 2007; Liu et al., 2012; Novák et al., 2012; Porfírio et al., 2016; Pěnčík et al., 2018; Wang et al., 2020). Until now, a wide range of sorbents have been used for purification: reverse phase columns C18 (Rolčík et al., 2005; Tivendale et al., 2012), and HLB (Novák et al., 2012), poly(styrene-divinylbenzene) copolymer SDB-XC (Pěnčík et al., 2018) or mixed-mode ion-exchange polymeric sorbents (Dobrev et al., 2005; Izumi et al., 2009). Gas chromatography and high-performance liquid chromatography (HPLC) are the most widely used methods for auxin detection (Porfírio et al., 2016). Modern methods provide fast and efficient separation of several classes of phytohormones (Šimura et al., 2018; Široká et al., 2022). Examples given are nanoflow capillary liquid chromatography (Izumi et al., 2009) or ultra-high performance liquid chromatography using sub-2 μm particles and higher pressure tolerance (1000 bar versus 500 bar for HPLC), which together allow more efficient and faster separation of substances (Novák et al., 2008; Kojima et al., 2009). For auxin analysis, the combination of high-performance liquid chromatography with tandem mass spectrometry (HPLC-MS/MS) is currently the most used method (Kai et al., 2007a; Novák et al., 2012; Sugawara et al., 2015; Pěnčík et al., 2018).

Although some oxIAA-amino acids have been identified in plants, information about their occurrence in various plant species and distribution within individual plant organs is still under-investigated. In this study, we identified two novel oxIAA amide conjugates oxIAA-Leu and oxIAA-Phe and performed tissue-specific quantitative determination of four 2-oxindole-3-acetic acid amides: oxIAA-Asp, oxIAA-Glu, oxIAA-Leu and oxIAA-Phe using optimised protocol combining micro-scale in-tip solid-phase extraction (in-tip μSPE) with HPLC-MS/MS. In order to uncover the diversity of IAA metabolism, we further analysed IAA metabolite profiles of four representative angiosperm plant models - *Arabidopsis thaliana*, pea (*Pisum sativum* L.), wheat (*Triticum aestivum* L.) and maize (*Zea mays* L.) at multiple growth stages according to the Biologische Bundesanstalt, Bundessortenamt und Chemische Industrie (BBCH) scale (Tottman, 1987; Lancashire et al., 1991; Boyes et al., 2001).

## Materials and methods

### Reagents and standards

The standards for IAA and indole-^13^C_6_-labeled IAA were purchased from Merck (Germany). Standards for IAA-Asp, IAA-Glu, IAA-Leu, IAA-Phe, oxIAA, and ^13^C_6_-[benzene ring]-IAA-Asp, [^13^C_6_]IAA-Glu and [^13^C_6_]oxIAA were purchased from OlChemIm (Czech Republic). IAA-glc, oxIAA-glc, [^13^C_6_]IAA-glc, [^13^C_6_]oxIAA-glc, oxIAA-Asp, oxIAA-Glu, oxIAA-Leu, oxIAA-Phe, oxIAA-[^13^C_4_,^15^N]Asp, oxIAA-[^13^C_5_,^15^N]Glu were synthesised in accordance to (Kai et al., 2007a; Kai et al., 2007c) with minor modifications. Plant agar and Murashige & Skoog media were purchased from Duchefa; methanol for HPLC analysis and all other chemicals were purchased from Merck (Germany) and Lach-Ner (Czech Republic).

### Plant material and growth conditions

Arabidopsis seeds (ecotype Col-0) were sterilized with 70% ethanol with the addition of 0.1% Tween 20 for 5 minutes, sown on Murashige & Skoog square agar plates (10 g sucrose, 4.4 g MS medium, 10 g agar per liter, pH 5.7) and cold treated for 4 days prior to germination. The plates were then transferred to a cultivation chamber and vertically placed under long-day conditions (16 h light/8 h dark) at 22 ± 1°C under cool white fluorescent light (maximum irradiance 550 μmol m^−2^ s^−1^). The seedlings were harvested at different growth stages according to the BBCH scale (**Table S1**; **Figure S1**). Shoots and roots were separated and weighed in five replicates, homogenized and immediately frozen in liquid nitrogen, and stored at -80°C until extraction.

Pea (*Pisum sativum arvense* L.), wheat (*Triticum aestivum* L.), and maize (*Zea mays* L.) seeds were left germinating in the dark for two, three and four days, respectively, selected according to their uniformity from a large population, transferred to hydroponic boxes, watered with Hoagland’s solution and left growing in the cultivation room (16 h light/8 h dark) at 22 ± 2°C. Three to five plants per growth stage were harvested (**Table S1**; **Figure S1**). Shoots, roots, and cotyledons were separated and weighed in five replicates, homogenized and immediately frozen in liquid nitrogen, and stored at -80°C until extraction.

### Extraction and purification of auxins

Frozen samples containing 10 mg of fresh weight (pea, maize, wheat, Arabidopsis shoot) or 3 mg (Arabidopsis root) were extracted in 1 ml (500 µl for Arabidopsis root samples) of ice-cold Na-phosphate buffer (50 mM, pH 7.0, 4°C) containing 0.1% diethyldithiocarbamic acid sodium salt. A mixture of stable isotope-labeled internal standards ([^13^C_6_]IAA, [^13^C_6_]IAA-Asp, [^13^C_6_]IAA-glc, [^13^C_6_]IAA-Glu, [^13^C_6_]oxIAA, oxIAA-[^13^C_4_,^15^N]Asp, [^13^C_6_]oxIAA-glc, oxIAA-[^13^C_5_,^15^N]Glu) was added to each sample in amount of 5 pmol each (2.5 pmol for Arabidopsis roots). Due to the high concentration of oxIAA-Asp in pea seeds, five replicates were diluted in an ice-cold Na-phosphate buffer by one hundred (10 µl of sample to 990 µl of Na-phosphate buffer) and then 5 pmol of internal standards were added as described above. All samples were then homogenized using a MM400 bead mill (Retsch GmbH, Germany) at a frequency of 27 Hz for 10 minutes after adding three zirconium oxide beads. Extracted samples were incubated at 4°C with continuous shaking for 15 minutes and then centrifuged (15 minutes, 206 642 g, 4°C). From each sample, 200 µl of Na-phosphate buffer was transferred to clean microtubes, pH adjusted to 2.7 by 0.1 M hydrochloric acid and purified using in-tip μSPE (Pěnčík et al., 2018) with two types of extraction sorbents (three layers of each): HLB AttractSPE™ (Affinisep, France) and SDB-XC Empore™ (3M, USA). Briefly, multi-StageTip microcolumns were activated by 50 µl of acetone (centrifugation 10 minutes, 3 846 g, 8°C), 50 µl of methanol (10 minutes, 3 846 g, 8°C), and 50 µl of water (15 minutes, 4 654 g, 8°C). The acidified samples were then applied to activated microcolumns (centrifugation 30 minutes, 16 961 g, 8°C), washed by 50 µl of 0.1% acetic acid (20 minutes, 9 846 g, 8°C) and eluted with 50 µl of 80% methanol (20 minutes, 8 653 g, 8°C). After elution, the samples were evaporated to dryness *in vacuo*, and stored at -20°C until HPLC-MS/MS analysis.

For optimization of in-tip μSPE method, three types of extraction sorbents (SDB-XC Empore™, HLB AttractSPE™, and C18 Empore™) and their combinations were tested. For the single sorbent, five layers of HLB or SDB-XC were applied to the StageTip microcolumn. For multiple sorbent combinations, three layers of each sorbent were used. To compare extraction recovery of each microcolumn, 200 µl of acidified Na-phosphate buffer containing 2 pmol of unlabeled standards (IAA, IAA-Asp, IAA-glc, IAA-Glu, oxIAA, oxIAA-Asp, oxIAA-glc, oxIAA-Glu, oxIAA-Leu, oxIAA-Phe) was applied to activated microcolumns, washed and eluted as mentioned above. The samples were then evaporated to dryness *in vacuo* and stored at -20°C until HPLC-MS/MS analysis. Finally, process efficiency of each sorbent was expressed as a percentage recovery and calculated as the ratio of the peak area of an unlabeled analyte spiked before μSPE to the peak area of the neat solution (Matuszewski et al., 2003).

### Quantification of IAA metabolites

The evaporated samples were dissolved in 30 μl of 10% methanol prior to HPLC-MS/MS analysis using a 1290 Infinity LC system and a 6490 Triple Quadrupole LC/MS system equipped with Jet Stream and Dual Ion Funnel systems (Agilent Technologies, CA, USA). The chromatographic reverse-phase column (Kinetex C18 100A, length 50 mm, diameter 2.1 mm, particle size 1.7 μm; Phenomenex, CA, USA) was used for the separation of individual analytes by HPLC. The mobile phase consisted of deionized water (A) and methanol (B) with the addition of 0.1% acetic acid. The time of each analysis was 18 min, flow rate 0.3 ml/min and IAA metabolites were eluted using gradient elution as follows: 0 min – 10% B, 11.5 min – 60% B, 11.75 min – 100% B, 14.75 min – 100% B, 15 min – 10% B. For IAA-glc quantification in Arabidopsis, different type of column (Kinetex C18 100A, length 150 mm, diameter 2.1 mm, particle size 1.7 μm; Phenomenex, CA, USA) was used with the identical chromatographic conditions as described above. During analysis, the samples were stored in an autosampler at 4°C, a column tempered at 40°C and 10 μl of each sample was injected.

Individual analytes were detected by the MS instrument combining positive and negative ESI mode (+/-) with optimised conditions as follows: nebulizer pressure, 25 psi; drying gas flow and temperature, 14 l/min and 130°C; sheath gas flow and temperature, 12 l/min and 400°C; capillary voltage, 2.8 kV in positive mode and 3.0 kV in negative mode; nozzle voltage, 0 V. Analytes were detected and quantified using diagnostic multiple reaction monitoring (MRM) transitions of precursor and appropriate product ions using optimal collision energies and 50 ms dwell time (**Table S2**). For data interpretation, Mass Hunter software (Agilent Technologies, CA, USA) was used.

### Method validation

Seven-day-old pea and wheat seedlings were harvested, weighed, and immediately frozen in liquid nitrogen, and stored at -80°C until extraction. Samples of 10 mg of FW were extracted in 1 ml of Na-phosphate buffer (50 mM, pH 7.0, 4°C, 0.1% diethyldithiocarbamic acid sodium salt) as previously described. After centrifugation, the samples were diluted to 5 ml with Na-phosphate buffer and each sample divided into doses of 200 µl (15 samples per plant). Subsequently, in five replicates, samples were supplemented with 1 pmol, 10 pmol or without unlabeled standards (IAA, IAA-Asp, IAA-glc, IAA-Glu, oxIAA, oxIAA-Asp, oxIAA-glc, oxIAA-Glu, oxIAA-Leu, oxIAA-Phe) and 5 pmol of internal stable isotope-labeled standards ([^13^C_6_]IAA, [^13^C_6_]IAA-Asp, [^13^C_6_]IAA-Glu, [^13^C_6_]IAA-glc, [^13^C_6_]oxIAA, oxIAA-[^13^C_4_,^15^N]Asp, [^13^C_6_]oxIAA-glc, oxIAA-[^13^C_5_,^15^N]Glu). All samples were then acidified and purified by in-tip μSPE method as described above. Finally, concentrations of all analytes were quantified by HPLC-MS/MS using a standard isotope dilution method. To calculate the accuracy of the method, the deviation of the determined analyte concentration (after subtraction of endogenous level) from its nominal level (known amount of analyte standard added to sample) was divided by the nominal level and expressed as percentage bias. Method precision was calculated as the relative standard deviation of determined levels.

### Data analysis

Multivariate data analysis was performed using SIMCA 17 software (Sartorius Stedim Data Analytics, Umeå, Sweden). Dataset containing endogenous concentrations (pmol/g FW) was log transformed, centered and scaled using unit-variance scaling method. Values under limit of detection (LOD) were replaced with 0.95-fold of respective LOD value. Principal component analysis (PCA) was calculated for description of data structure. All PCA models were cross-validated to assess model reliability. Variables with no or neglectable effect in score plot were excluded.

## Results

### Development and validation of oxIAA-amino acids profiling method

Solid phase extraction is becoming the most widely used method for the purification of phytohormones. In the last decade, there has been a trend towards minimal use of solvents, which has led to the development of new solid phase microextraction such as in-tip µSPE, which was first used for cytokinin analysis (Svačinová et al., 2012) and later for auxin purification (Liu et al., 2012; Pěnčík et al., 2018). We adopted the in-tip µSPE purification protocol described by Pěnčík et al. (2018) to achieve the highest possible extraction efficiency for all IAA metabolites including novel oxIAA-AA conjugates. The recovery rate of analytes was tested using three types of extraction sorbents and their combination: SDB-XC Empore™, HLB AttractSPE™, and C18 Empore™. The recoveries of added auxin standards in acidified Na-phosphate buffer applied onto microcolumns are summarized in **Figure S2**. The single-StageTips filled with HLB or SDB-XC sorbents recovered 49 ± 9% and 59 ± 11% of auxin compounds, respectively. In good agreement with previously published data (Pěnčík et al., 2018), multi-StageTips C18/SDB-XC also showed high extraction yields (mean recovery 60 ± 11%). Under our experimental conditions, the HLB/SDB-XC microcolumns combined hydrophilic-lipophilic balance/polymer-based sorbents provided the highest recoveries ranging from 47% to 89% across all analytes (**Figure S2**). Importantly, oxIAA-AA conjugates were efficiently retained on HLB/SDB-XC sorbents with recovery rates ranging from 47 ± 14% (oxIAA-Asp) to 76 ± 5% (oxIAA-Leu). According to our findings, a combination of HLB/SDB-XC sorbents was used to isolate oxIAA-AA conjugates from the studied plant material.

**Figure 2:**
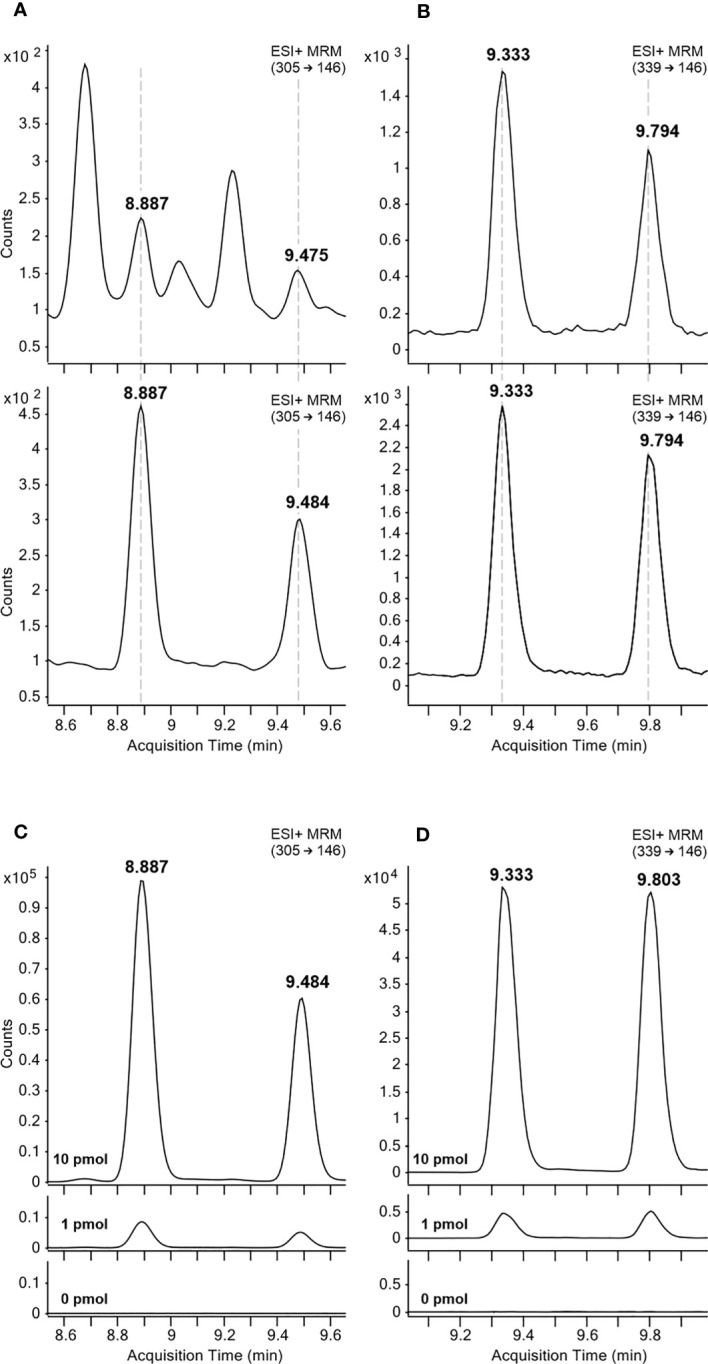
Representative MRM chromatograms of oxIAA-Leu **(A, C)** and oxIAA-Phe **(B, D)**. The retention times of oxIAA-Leu **(A)** and oxIAA-Phe **(B)** in pea cotyledons extracts containing 2 mg FW of the tissue (upper) and 5 fmol (oxIAA-leu) or 50 fmol (oxIAA-Phe) of synthetic reference standards (lower). Arabidopsis extracts spiked with 1 or 10 pmol of oxIAA-Leu **(C)** and oxIAA-Phe **(D)** synthetic standards.

All auxin metabolites were analysed by LC-MS/MS method using electrospray ionization in positive and negative modes. MRM transitions and collision energies were optimised for all analytes and their corresponding stable isotope labelled internal standards (**Table S2**). Confirmation of the newly identified metabolites in pea cotyledons was performed based on comparisons to the retention times of a synthetic oxIAA-AA reference standard (**Figure 2**). Importantly, two chromatographic peaks were detected due to the optical activity of investigated compounds at position three in the oxindole molecule, 8.9/9.5 min for oxIAA-Leu and 9.3/9.8 min for oxIAA-Phe. Moreover, each sample extract (Arabidopsis, wheat and pea) was spiked with a mixture of oxIAA-Leu and oxIAA-Phe at known levels (0, 1.0 and 10.0 pmol). These results also confirmed the detection of two chromatographic peaks of both new amino acid conjugates. Representative MRM chromatograms of the spiked Arabidopsis samples are shown in **Figure 2**.

For method validation, 18-point calibration curves ranging from 45 amol to 90 pmol were constructed and the LOD (S/N ratio > 3) as well as the dynamic linear range were calculated (**Table S2**). For newly identified metabolites, the linear range stretched from 9 fmol to 90 pmol, and for the other compounds, the linear range started even at 90 amol. Finally, a spiking experiment with authentic standards of auxin metabolites was performed. Wheat and pea extracts were used for method validation as representatives of monocots and dicots, respectively. A standard mixture containing 1 or 10 pmol of individual authentic standards was added to extracts containing 2 mg of plant material (homogenized whole plants) and their recovery was calculated after subtraction of endogenous levels (Matuszewski et al., 2003). The method accuracy (percentage deviation from the accepted reference value, % bias) and method precision (relative standard deviation of determined levels, RSD) were then calculated (**Table S3**). Mean method accuracy was 15 ± 12% bias for pea samples and 19 ± 15% bias for extracts prepared from wheat seedlings. Method precisions of all analytes were below 14% RSD for both tested matrices (**Table S3**). Overall, our results showed that the method has good linearity, high sensitivity and sufficient precision and accuracy, and thus the levels of auxin metabolites were finally determined.

### Quantification of oxIAA-amino acid conjugates

**Figure 3:**
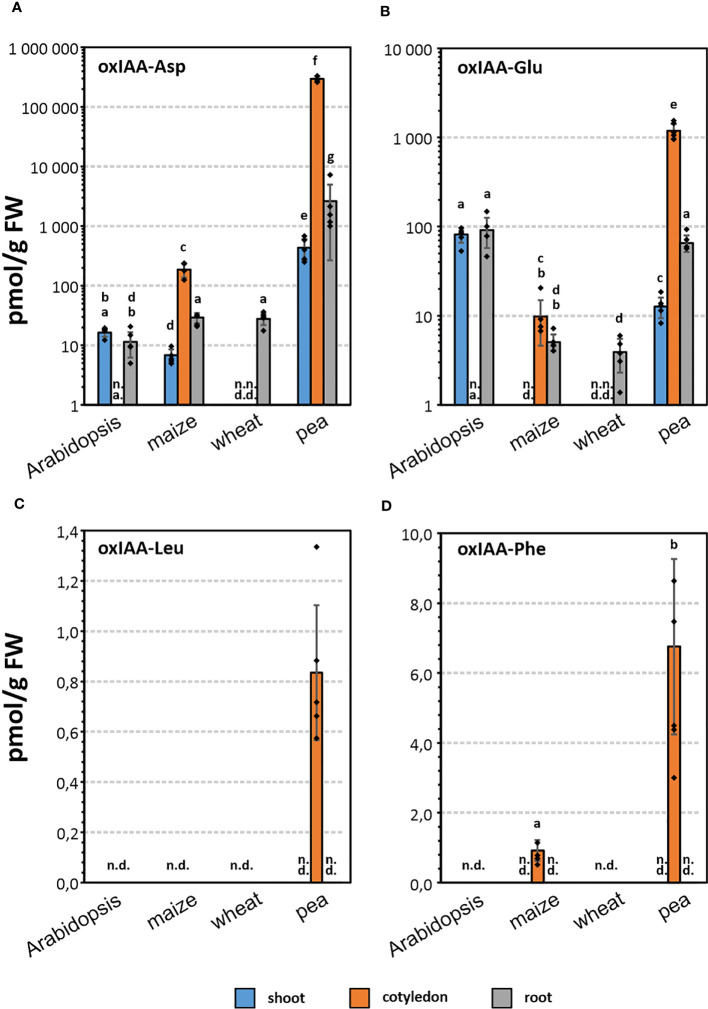
Endogenous levels (pmol · g^-1^ FW) of oxIAA-Asp **(A)**, oxIAA-Glu **(B)**, oxIAA-Leu **(C)** and oxIAA-Phe **(D)** in root, shoot, and cotyledon of maize, wheat, pea, and root and shoot of Arabidopsis. Levels of oxIAA-Asp and oxIAA-Glu are shown on a logarithmic scale. The error bars indicate the standard deviations of five replicates (mean ± SD, n=5). One-way ANOVA and Tukey’s post hoc test were applied to log-transformed data to assess the differences between groups. Different letters (a-g) indicate significant differences at the 5% level of significance. n.d., not detected; n.a., not analysed.

Endogenous levels of oxIAA-AAs were determined in roots, cotyledons and shoots of three crop species (maize, pea, and wheat), and in shoots and roots of Arabidopsis (**Figure 3**; **Table S4**). In order to obtain comparable data, all species were harvested at the same growth stage 1.0 (**Table S1**; **Figure S1**).

Conjugates of oxIAA with Asp and Glu were determined in all four analysed species, but the levels varied greatly depending on the species and particular part of the plant. In general, oxIAA-Asp was found to be the most abundant oxIAA conjugate in pea tissues, for example concentrations close to 300 nmol·g^-1^ FW in cotyledons (**Table S4**). In contrast, oxIAA-Glu was the major oxIAA-AA conjugate in Arabidopsis. The determined levels of oxIAA-Glu were 81/92 pmol·g^-1^ FW in Arabidopsis shoots/roots, while oxIAA-Asp was detected in lower quantities (16/11 pmol·g^-1^ FW). In both members of the Poaceae family, wheat and maize, oxIAA conjugates with Asp and Glu were partially determined. No oxIAA-Glu was detected in maize shoot samples (**Figures 3B**). Moreover, both amino acid conjugates were below the limit of detection in wheat shoots and cotyledons. Interestingly, endogenous levels of oxIAA-Asp showed similar pattern in maize tissues compared to pea samples, but at picomolar levels (**Figure 3A**).

The newly identified metabolites, oxIAA-Leu and oxIAA-Phe, were detected only in cotyledon samples, oxIAA-Phe in maize and both conjugates in pea (**Figures 3C, D**). Importantly, these Leu and Phe conjugates were much less abundant compared to oxIAA-Asp or oxIAA-Glu. Pea cotyledons contained 7 pmol·g^-1^ of oxIAA-Phe and less than 1 pmol·g^-1^ of oxIAA-Leu, similar to oxIAA-Phe level in maize cotyledons (**Table S4**).

### Metabolite profiles of IAA and oxIAA conjugates in different plant species

**Figure 4:**
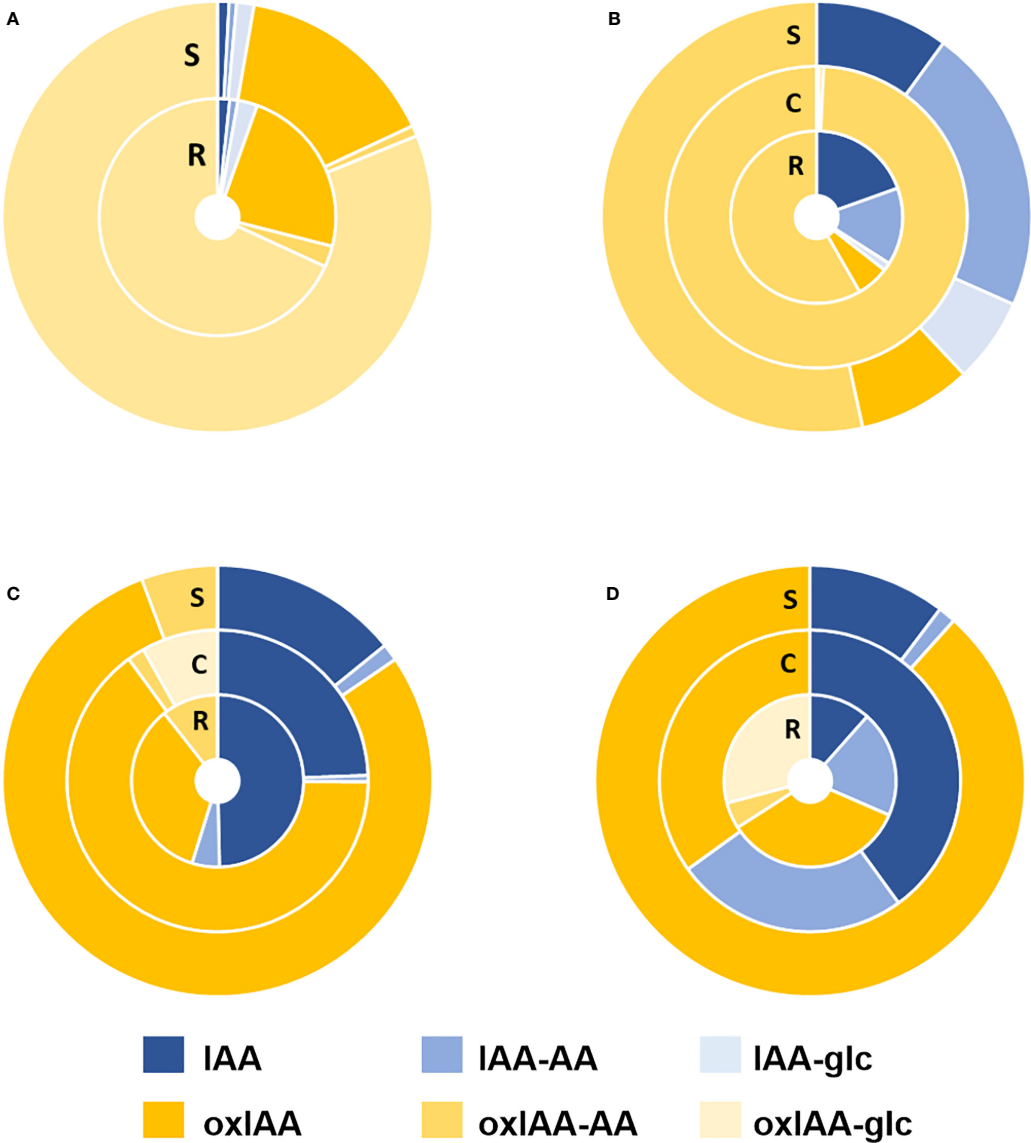
Relative distribution of auxin metabolites in Arabidopsis **(A)**, pea **(B)**, maize **(C)** and wheat **(D)** plants at 1.0 growth stage. IAA-AA represents a sum of IAA-Asp, IAA-Glu, IAA-Leu, and IAA-Phe relative abundances; oxIAA-AA represents a sum of oxIAA-Asp, oxIAA-Glu, oxIAA-Leu, and oxIAA-Phe relative abundances. Non-oxidized forms of IAA are shown in shades of blue and oxidized forms in yellow shades. All samples were measured in five replicates. R, Roots; S, Shoots; C, cotyledons.

Full auxin metabolite profiles including free IAA, oxIAA and their low-molecular-weight conjugates with amino acids and glucose were determined in the same four plant models at growth stage 1.0. The levels of IAA and oxIAA conjugates with individual amino acids (Asp, Glu, Leu and Phe) were summed up into two corresponding groups IAA-AA and oxIAA-AA, respectively. The contributions of different conjugate classes as well as glucosyl esters were calculated as their relative abundances to the total pool of IAA metabolites (**Figure 4**; **Table S5**). Overall, the relative distribution of auxin metabolites differed notably within studied species as well as within the individual plant parts.

The dominant conjugate in Arabidopsis was oxIAA-glc taking 81% (shoots) and 68% (roots) of all measured metabolites, while IAA-glc and amino acid conjugates of both IAA and oxIAA, as well as free IAA, were found to have a minor representation in Arabidopsis. The second most abundant metabolite was oxIAA, which took 15% of the IAA metabolite pool in the shoots and 23% in the roots (**Figure 4A**). In contrast, the major auxin metabolites in pea were oxIAA-AA conjugates, accounting for 53% in shoots and 58% in roots. IAA-AAs were the second most abundant conjugates in pea shoots (22%) and roots (15%). In cotyledons, oxIAA-AAs were fully dominant, taking 99% of all auxin metabolites (**Figure 4B**). Similar profiles of auxin metabolites were observed in maize and wheat. In both species, oxIAA was major metabolite in shoots (79% in maize; 88% in wheat). In other parts, oxIAA was also the most abundant inactive auxin metabolite; only free IAA had a greater proportion in maize roots and wheat cotyledons. Amide conjugates of oxIAA were also present in maize and wheat, but occupied a relatively minor part of the total auxin metabolite profile (**Figures 4C, D**).

### Spatiotemporal dynamics of auxin metabolites distribution during early plant development

We further investigated the distribution of IAA and its metabolites during the early development of Arabidopsis, pea, maize and wheat. Therefore, different plant parts such as roots, shoots (all species) and cotyledons (only crop species) were sampled at different growth stages. The first analysed stage was 1.0, in which the first leaf emerges from the coleoptile in grains, or cotyledons are fully unfolded in pea and Arabidopsis. The following stages were 1.1 and 1.2, in which the first and second leaf are fully developed and unfolded (**Figure S1**). However, the stage 1.1 is not differentiated in Arabidopsis as first two leaves develop simultaneously.

**Figure 5:**
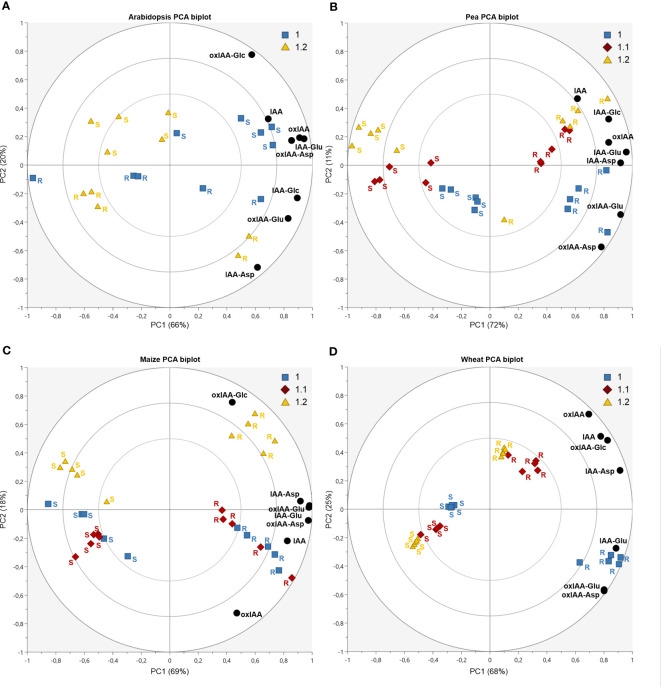
Auxin metabolites distribution dynamics during plant development in Arabidopsis **(A)**, pea **(B)**, maize **(C)** and wheat **(D)**. Each figure displays a PCA biplot, axes PC1 and PC2 express correlation-scaled loadings as concentration of metabolites (black circles) and correlation-scaled scores as samples of root (R) and shoot (S) at growth stages 1.0 (blue squares), 1.1 (red diamonds) and 1.2 (yellow triangles).

**Figure 6:**
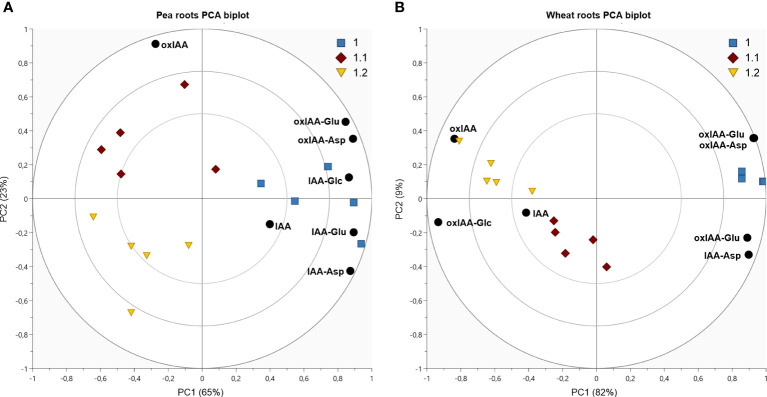
Dynamics of auxin metabolites distribution in pea shoots **(A)** and wheat roots **(B)**. Both figures display PCA biplot, axes PC1 and PC2 express correlation-scaled loadings as concentration of metabolites (black circles) and correlation-scaled scores as samples at growth stages 1.0 (blue squares), 1.1 (red diamonds) and 1.2 (yellow triangles).

Principal component analysis (PCA) was used to observe the variability in auxin metabolite content. As already indicated in **Figure 4**, the significant differences of the relative distribution of auxin metabolites between plant species was observed. Therefore, each species was evaluated in individual PCA model to describe the distribution of metabolites in roots and shoots and growth stages (**Figure 5**, complete dataset provided as **Supplementary Table 6**). The most complex patterns found in this study were later analysed using additional PCA to describe changes in the development of individual plant parts (**Figure 6**).

In Arabidopsis, only stages 1.0 and 1.2 were analysed. At stage 1.0 the cotyledons are completely opened, while stage 1.2 is defined by fully developed first two true leaves (Boyes et al., 2001). A more pronounced stage-dependent alteration of auxin metabolome was observed in shoots, where auxin metabolite content was higher at the early stage (1.0) and dropped during seedling development up to stage 1.2 (**Figure 5A**). This trend was observed not only in auxin metabolites, but also in free active IAA. Interestingly, there were no significant changes in auxin metabolome in the roots during development. Regardless of the stages, oxIAA-glc levels were much higher in shoots, compared to roots. Pea roots contained a higher concentration of auxin metabolites than shoots at all growth stages (**Figure 5B**). The roots at stage 1.0 were characterised by the highest content of oxIAA-Asp and oxIAA-Glu, the concentrations of which gradually decreased until stage 1.2. A similar trend was observed in the shoots, where the highest levels of all IAA and oxIAA conjugates were determined at stage 1.0. The levels of conjugates dropped towards later stages, while oxIAA showed a maximum at middle stage 1.1 (**Figure 6A**). In maize and wheat, higher concentrations were also observed in roots compared to shoots throughout the development period. In maize, the two earlier stages were associated with higher levels of oxIAA, while the last stage was characterized by the accumulation of oxIAA-glc in roots (**Figure 5C**). With the increasing stage of development, the concentration of amino acid conjugates of both IAA and oxIAA decreased in wheat roots, while oxIAA and oxIAA-glc levels have changed in the opposite manner (**Figures 5D**, **6B**).

## Discussion

Auxin metabolism is a complex process, which is still not very well understood. Several mechanisms of IAA inactivation and degradation have been described so far. One of the most intensively studied and best characterised mechanisms of IAA inactivation is the conjugation with amino acids mediated by GH3 enzyme family (Staswick et al., 2005). The amides of IAA can be then irreversibly oxidized by DAO1 to form oxIAA-AAs (Müller et al., 2021). Hayashi et al. (2021) revealed that in Arabidopsis, oxIAA-AAs are hydrolysed by amido hydrolases to oxIAA, which is then possibly further oxidized to 3-hydroxy-oxIAA (dioxIAA). According to the newly proposed model of auxin metabolism, conjugation with amino acids and oxidation of indole ring contribute to the same pathway, in which oxIAA-AAs serve as intermediates (**Figure 1**). This pathway involving oxIAA-AAs is proposed as a main route of IAA inactivation and is therefore a key mechanism contributing to auxin homeostasis in Arabidopsis (Hayashi et al., 2021). Interestingly, it was recently shown that the GH3-ILR1-DAO pathway does not operate in non-flowering plants (Brunoni et al., 2020; Brunoni et al., 2023). Apart from Arabidopsis, endogenous levels of oxIAA-Asp and oxIAA-Glu were recently determined in lychee (Kim et al., 2021; Osako et al., 2022), oxIAA-Asp also in rice (Isobe and Miyagawa, 2022). However, there is no evidence about their endogenous levels and distribution in other plant species. Furthermore, the occurrence of oxIAA conjugates with other amino acids was not yet reported.

In our study, we focused on investigating the abundance and distribution of oxIAA amino acid conjugates (oxIAA-Asp/Glu/Leu/Phe) in Arabidopsis and three important crop species: maize, pea and wheat. Therefore, we utilized an optimised analytical method combining micro-scale purification with LC-MS/MS. For the isolation of oxIAA amino acid conjugates, we adopted the µSPE protocol previously developed for the purification IAA metabolites (Pěnčík et al., 2018). To maximise the yield of the purification step, we compared the recoveries of oxIAA-AAs as well as other IAA metabolites using various types of SPE sorbents and their combinations. Finally, we used multi-µSPE columns assembled from a combination of HLB and SDB-XC sorbents, which provided the highest overall extraction yield of all tested sorbents (**Figure S2**). The accuracy and precision of the analytical method was then tested by quantifying known amounts of authentic standards added to pea and wheat extracts. Overall, the method validation demonstrated good applicability of the new protocol for the quantitative analysis of auxin metabolites in distinct plant matrices (**Table S3**). Unsatisfactory method accuracy (up to 48% bias) was achieved only for oxIAA-Leu and oxIAA-Phe, for which corresponding isotopically labelled standards are still missing. Unfortunately, the close-eluting internal standard oxIAA-[^13^C_5_,^15^N]Glu was not able to fully compensate for analytes losses during sample processing and subsequent LC-MS/MS analysis. Importantly, the reduced accuracy was mainly detected in wheat samples in which the two novel naturally occurring auxin metabolites were not detected (**Figures 3C, D**).

The optimised and validated analytical method was then utilized for determination of oxIAA amino acid conjugates in Arabidopsis and three crop species: pea, maize and wheat. In Arabidopsis, oxIAA-Asp and oxIAA-Glu were determined in comparable levels in both roots and shoots. According to previously performed quantification (Hayashi et al., 2021), oxIAA-Glu was the most abundant conjugate in Arabidopsis, in contrast to other studied species, where oxIAA-Asp dominated throughout all seedling parts. In crop species, oxIAA conjugates were mainly accumulated in cotyledons, except for wheat, in which oxIAA conjugates were detected only in roots. While oxIAA-Asp and oxIAA-Glu were found in all studied species, oxIAA-Leu was detected only in pea cotyledons, as well as oxIAA-Phe, which was additionally detected in cotyledons of maize. Kai et al. (2007a) previously observed formation of oxIAA-Phe in Arabidopsis after administration of IAA-Phe. However, here we report for the first time oxIAA conjugates with Leu and Phe as endogenous plant metabolites.

The endogenous content of oxIAA amino acid conjugates was then evaluated in the context of overall auxin metabolome. In accordance with previous studies (Kai et al., 2007a; Pěnčík et al., 2018), oxIAA-glc was determined as a major auxin metabolite in Arabidopsis, followed by oxIAA, while amide-linked conjugates occupied relatively minor part of the total pool (**Figure 4A**). In strong contrast to Arabidopsis, surprisingly no oxIAA-glc was detected in pea, whereas oxIAA amino acids represented the dominant class of metabolites in both roots and shoots. In pea cotyledons, they even made over 99% of total auxin metabolite content (**Figure 4B**). Our results are consistent with previous findings that the majority of IAA in pea is present as low or high molecular weight amide conjugates (Bandurski and Schulze, 1977). Interestingly, IAA-glc was below the limit of detection in all sections of maize and wheat seedling (**Figures 4C, D**). Nevertheless, IAA esters were found to be the main conjugated auxin form in seeds of both crops, as well as in vegetative tissue in case of maize (Bandurski and Schulze, 1977). Most likely, a major part of ester-linked auxin is represented by high molecular weight conjugates or indole-3-acetyl-*myo*-inositol, which was reported to serve as an important storage form of auxin in maize seeds (Nowacki and Bandurski, 1980).

Plant development is an ever-changing process controlled by phytohormones, the levels of which are being strictly regulated by *de novo* biosynthesis and metabolism during the entire period of plant growth. Therefore, we addressed the changes in concentrations and distribution of auxin and its metabolites within the seedling at early growth stages as defined by BBCH scale (Lancashire et al., 1991). During seedling development, the levels of auxin metabolites, including oxIAA-Asp and oxIAA-Glu, dropped in Arabidopsis shoots between stage 1.0, when the shoot is constituted of the hypocotyl and fully developed cotyledons, and stage 1.2, already having first pair of true leaves fully developed (**Figure 5A**). Interestingly, Ljung et al. (2001) showed that IAA levels in cotyledons and hypocotyls are relatively low and constant during early seedling development, while its content was dramatically higher in first two developing true leaves and decreased dramatically during following leaf expansion. Furthermore, high IAA levels were strongly correlated with high cell division rates in Arabidopsis leaves. It can be assumed that seedling at stage 1.0 already comprise primordia of the first pair of true leaves, in which the cells divide intensively and thus accumulate high levels of auxin. The change in auxin metabolite levels between growth stages 1.0 and 1.2 that we observed is most likely a consequence of the decrease in IAA concentration during true leaves development. In agreement with our results, a reduction of IAA metabolite levels during early Arabidopsis development was previously observed in one- or two-week-old seedlings (Tam et al., 2000; Kai et al., 2007a).

Consistently in all crops, higher concentrations of all metabolites were found in roots at all analysed growth stages (**Figures 5B-D**). A similar distribution was previously observed in another important crop, rice (*Oryza sativa* L.), where IAA amino acid conjugates (Matsuda et al., 2005; Kai et al., 2007b), as well as oxIAA-Asp, dioxIAA-Asp and dioxIAA-Glu (Isobe and Miyagawa, 2022) were determined at higher levels in roots compared to aerial parts. Like Arabidopsis, the levels of IAA metabolites also depended on growth stage in crop species. As a common trend across all three crop species, amide-linked conjugates were the most abundant at the earliest stage and decreased during the growth towards later stages. A very pronounced correlation between oxIAA-amino acids content and growth stage was observed in pea, where the levels of oxIAA-Asp and oxIAA-Glu were the highest at stage 1.0 and declined towards the stage 1.2 in both roots and shoots (**Figures 5B**, **6A**). Interestingly, oxIAA-glc displayed the opposite pattern to amide-linked conjugates in the roots of both monocots, as it accumulated mainly at the stage 1.2.

In summary, we performed a comprehensive profiling of auxin metabolites in Arabidopsis and three crop plant models (pea, maize and wheat) using an optimised and validated micro-scale purification protocol combined with LC-MS/MS method. The novel approach is suitable for profiling of broad range of auxin metabolites in various plant models. We also identified new endogenous auxin metabolites (oxIAA-Leu and oxIAA-Phe) in maize and pea cotyledons. Our findings showed that auxin metabolite profiles differed between species and individual parts of the seedling (root, shoot and cotyledon). Moreover, the levels of auxin metabolites also strongly depended on the growth stage of the seedling, suggesting an important role of IAA metabolism in maintaining the phytohormonal balance during plant development.

## Data availability statement

The original contributions presented in the study are included in the article/**Supplementary Material**. Further inquiries can be directed to the corresponding author.

## Author contributions

ON and AP conceived the project. PH and AP performed method development and optimization. PH grew and sampled the plants. PH conducted the purification and quantification of auxin metabolites. AZ synthesized all new oxIAA conjugates standards. PH, IP, ON and AP analysed and interpreted the data. PH prepared the manuscript draft. PH, ON and AP wrote the article with input from all authors. All authors contributed to the article and approved the submitted version.

## Supplementary material

Supplementary Table 6 Source dataset for the PCA analysis (Figures 5, 6).
