# Supplementary material for: Metabolic profiles of 2-oxindole-3-acetyl-amino acid conjugates differ in various plant species
Source: Front Plant Sci. 2023 Jul 18;14:1217421. doi: 10.3389/fpls.2023.1217421 (PMC10390838; doi:10.3389/fpls.2023.1217421)
Supplement: Supplementary Table 6 — Source dataset for the PCA analysis (Figures 5, 6). [file Presentation_1.pdf]

Supplementary Material

Metabolic profiles of 2-oxindole-3-acetyl-amino acid conjugates differ in various plant species

Pavel Hladík, Ivan Petřík, Asta Žukauskaitė, Ondřej Novák, Aleš Pěnčík*

*** Correspondence:** Corresponding Author: alespencik@seznam.cz

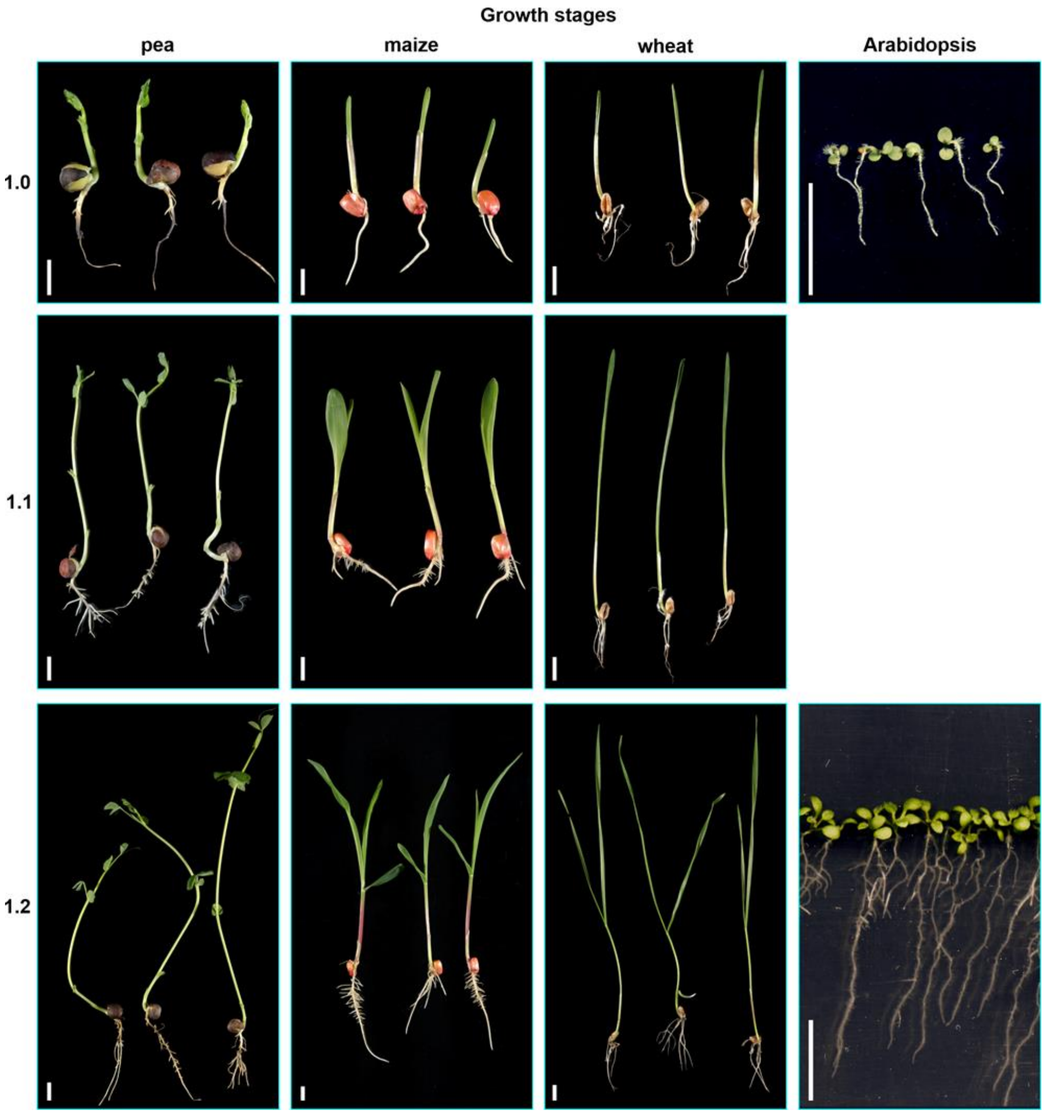

Supplementary Figure 1: Plant growth stages according to BBCH scale.
At stage 1.0, the cotyledons are fully unfolded in pea (A) and Arabidopsis (D), or the first leaf emerges from the coleoptile in maize (B) and wheat (C). The following stage 1.1 shows first leaf fully unfolded in pea (or first tendril developed) (E), maize (F) and wheat (G). At the last stage 1.2, two leaves are fully developed and unfolded in pea (or second tendril developed) (H), maize (I), wheat (J) and Arabidopsis (K). Scale bars indicate 1 cm.

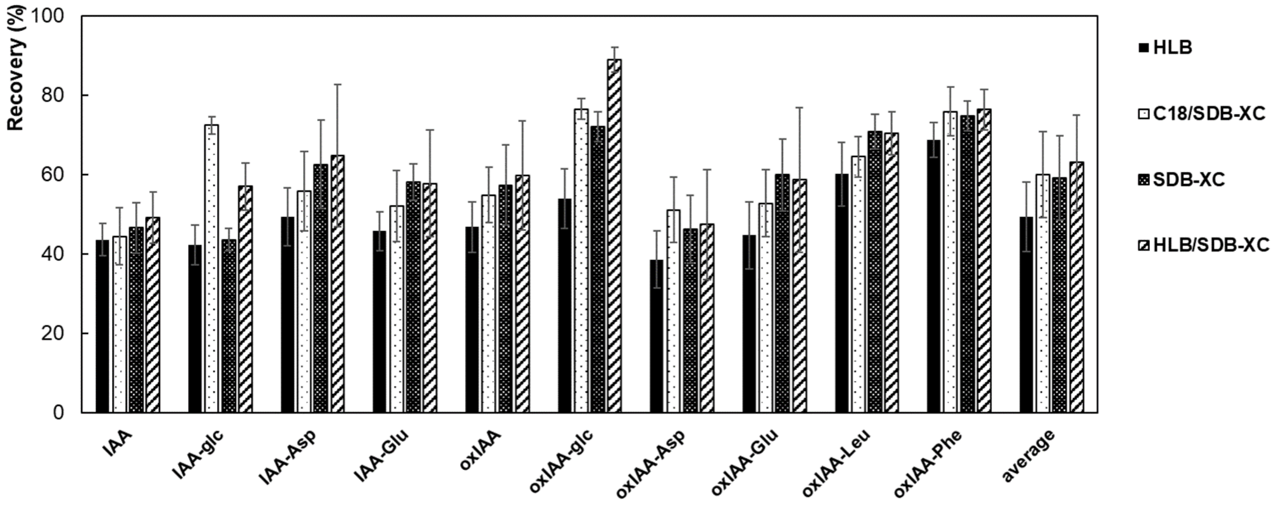

Supplementary Figure 2: In-tip μSPE sorbents optimization.
Recoveries (%) of unlabelled auxin standards (2 pmol added to extraction solvent) were calculated as a recovery of metabolite initial amounts. For all analytes, two types of extraction sorbents (SDB-XC Empore^{TM} and HLB AttractSPE^{TM}) or two multi-StageTips microcolumns (C18/SDB-XC and HLB/SDB-XC) were tested and then average recoveries were calculated. All sorbents' combinations were analysed in four replicates and error bars indicate standard deviations of the means (mean ± SD, n=4).
