# Supplementary material for: Metabolic profiles of 2-oxindole-3-acetyl-amino acid conjugates differ in various plant species
Source: Front Plant Sci. 2023 Jul 18;14:1217421. doi: 10.3389/fpls.2023.1217421 (PMC10390838; doi:10.3389/fpls.2023.1217421)
Supplement: Supplementary file 2 [file Table_1.pdf]

Supplementary Table 1: Age of plants harvested at different growth stages.
On the dedicated days after planting, four to six plants per species were harvested at the growth stages 1.0, 1.1, and 1.2 of the BBCH scale. For Arabidopsis, stage 1.1 is not differentiated as the first two leaves develop simultaneously.

Growth stages	Arabidopsis	Maize	Pea	Wheat
	(days)			
1.0	7	7	9	5
1.1	-	9	12	7
1.2	10	11	14	10
