# Supplementary material for: Metabolic profiles of 2-oxindole-3-acetyl-amino acid conjugates differ in various plant species
Source: Front Plant Sci. 2023 Jul 18;14:1217421. doi: 10.3389/fpls.2023.1217421 (PMC10390838; doi:10.3389/fpls.2023.1217421)
Supplement: Supplementary file 3 [file Table_2.pdf]

Supplementary Table 2: Conditions of HPLC-MRM-MS method.

Diagnostic MRM transitions and collision energies (CE) were optimised for all auxin metabolites and corresponding internal standards. In addition, retention time (RT), limit of detection (LOD), linear range and linearity (coefficient of determination, R^2) were also measured. Analytes were detected by the MS instrument combining positive and negative ESI mode (+/–) with optimised conditions as follows: nebulizer pressure, 25 psi; drying gas flow and temperature, 14 l/min and 130 °C; sheath gas flow and temperature, 12 l/min and 400 °C; capillary voltage, 2.8 kV in positive mode and 3.0 kV in negative mode; nozzle voltage, 0 V.

**Analyte**	**RT** (min)	**ESI**	**MRM**	**IS**	**IS MRM**	**CE** (V)	**LOD** (fmol)	**Linear range** (pmol)	R^2
IAA	5.6	+	176>130	[$^{13}C_6$]IAA	182>136	24	0.045	$9*10^{-5}$ - 90	0.9989
IAA-Asp	3.7	+	291>130	[$^{13}C_6$]IAA-Asp	297>136	36	0.45	$9*10^{-4}$ - 90	0.9992
IAA-Glu	4.4	+	305>130	[$^{13}C_6$]IAA-Glu	311>136	24	0.45	$4.5*10^{-4}$ - 90	0.9992
IAA-Leu	10.3	+	289>130	[$^{13}C_6$]IAA-Glu	311>136	36	0.9	$9*10^{-4}$ - 90	0.9980
IAA-Phe	10.7	+	323>130	[$^{13}C_6$]IAA-Glu	311>136	38	4.5	$4.5*10^{-3}$ - 90	0.9990
IAA-glc	4.2	–	336>174	[$^{13}C_6$]IAA-glc	342>180	8	90	$9*10^{-2}$ - 90	0.9988
oxIAA	3.9	+	192>146	[$^{13}C_6$]oxIAA	198>152	12	0.9	$9*10^{-4}$ - 90	0.9938
oxIAA-Asp	2.4; 2.7	+	307>146	oxIAA-[$^{13}C_4$,^{15}N]Asp	312>146	26	4.5	$9*10^{-3}$ - 90	0.9976
oxIAA-Glu	3.0; 3.3	+	321>146	oxIAA-[$^{13}C_5$,^{15}N]Glu	327>146	30	4.5	$9*10^{-3}$ - 90	0.9982
oxIAA-Leu	8.9; 9.5	+	305>146	oxIAA-[$^{13}C_5$,^{15}N]Glu	327>146	30	0.45	$9*10^{-4}$ - 90	0.9974
oxIAA-Phe	9.3; 9.8	+	339>146	oxIAA-[$^{13}C_5$,^{15}N]Glu	327>146	30	4.5	$9*10^{-3}$ - 90	0.9974
oxIAA-glc	3.2	–	352>190	[$^{13}C_6$]oxIAA-Glc	358>196	8	0.45	$4.5*10^{-4}$ - 90	0.9987
