# Supplementary material for: Metabolic profiles of 2-oxindole-3-acetyl-amino acid conjugates differ in various plant species
Source: Front Plant Sci. 2023 Jul 18;14:1217421. doi: 10.3389/fpls.2023.1217421 (PMC10390838; doi:10.3389/fpls.2023.1217421)
Supplement: Supplementary file 4 [file Table_3.pdf]

Supplementary Table 3: Method validation.

Method accuracy (% BIAS) and method precision (% RSD) were determined by spiking experiment. Briefly, samples were extracted in 1 ml of Na-phosphate buffer with 10 mg of pea or wheat homogenized plants, diluted to 5 ml with Na-phosphate buffer and each sample aliquoted into 200 µl doses (total 15 samples per plant). To each sample, 5 pmol of stable isotope-labelled standards were added. Subsequently, the samples were supplemented with unlabelled standards (1 or 10 pmol). The samples were then purified by in-tip µSPE method and the concentrations of each analyte were determined by HPLC-MS/MS using isotope dilution method. A set of plant extracts were also processed without the addition of unlabelled standards, and endogenous levels of auxin metabolites were subtracted before calculating the validation parameters. All samples were analysed in five replicates.

**Analyte**	**Pea**		**Wheat**	
	1 pmol	10 pmol	1 pmol	10 pmol
	BIAS (%) RSD (%)	BIAS (%) RSD (%)	BIAS (%) RSD (%)	BIAS (%) RSD (%)
IAA	5.7 / 4.8	14.0 / 7.3	16.2 / 6.3	14.1 / 2.6
IAA-Asp	16.3 / 13.8	7.5 / 6.1	24.5 / 4.5	8.5 / 4.4
IAA-Glu	31.8 / 6.1	0.1 / 4.3	33.6 / 4.1	2.6 / 3.5
IAA-Leu	36.4 / 2.8	21.7 / 7.9	30.4 / 2.7	17.2 / 2.3
IAA-Phe	29.1 / 4.2	11.5 / 4.2	18.5 / 0.7	3.3 / 1.4
IAA-glc	0.2 / 1.0	0.5 / 2.0	17.9 / 8.4	17.6 / 3.0
oxIAA	29.1 / 11.6	1.9 / 4.4	23.7 / 10.4	19.7 / 2.1
oxIAA-Asp	19.0 / 6.9	3.0 / 4.0	6.4 / 1.2	4.0 / 6.1
oxIAA-Glu	2.0 / 3.1	0.2 / 4.0	3.0 / 3.5	0.6 / 2.3
oxIAA-Leu	35.3 / 2.3	27.0 / 8.1	48.2 / 3.1	44.4 / 3.7
oxIAA-Phe	25.4 / 3.5	20.6 / 6.6	45.2 / 5.8	43.4 / 4.5
oxIAA-glc	21.2 / 3.5	7.4 / 4.3	11.2 / 6.8	0.3 / 5.3
