# Supplementary material for: Metabolic profiles of 2-oxindole-3-acetyl-amino acid conjugates differ in various plant species
Source: Front Plant Sci. 2023 Jul 18;14:1217421. doi: 10.3389/fpls.2023.1217421 (PMC10390838; doi:10.3389/fpls.2023.1217421)
Supplement: Supplementary file 5 [file Table_4.pdf]

Supplementary Table 4: Quantification of oxIAA-amino acid conjugates.
Four oxIAA-AAs were quantified (pmol/g FW ± SD; n=5) in roots, shoots and cotyledons of pea, wheat and maize, and roots and shoots of Arabidopsis. <LOD, under the limit of detection.

		**oxIAA-Asp**	**oxIAA-Glu**	**oxIAA-Leu**	**oxIAA-Phe**
			(pmol/g FW ± SD)		
Arabidopsis	shoot	16.3 ± 2.4	81.3 ± 15.3	<LOD	<LOD
	root	11.4 ± 5.2	91.5 ± 34.2	<LOD	<LOD
Maize	shoot	6.8 ± 1.7	<LOD	<LOD	<LOD
	cotyledon	186.7 ± 51.2	9.8 ± 5.2	<LOD	0.9 ± 0.3
	root	29.1 ± 5.7	5.1 ± 1.1	<LOD	<LOD
Wheat	shoot	<LOD	<LOD	<LOD	
	cotyledon	<LOD	<LOD	<LOD	<LOD
	root	27.8 ± 5.9	3.9 ± 1.6	<LOD	<LOD
Pea	shoot	436.0 ± 168.9	12.7 ± 3.3	<LOD	<LOD
	cotyledon	296 480.2 ± 27 479.2	1 194.2 ± 221.2	0.8 ± 0.3	6.8 ± 2.5
	root	2 618.2 ± 2 350.7	65.6 ± 13.7	<LOD	<LOD
