# Supplementary material for: Metabolic profiles of 2-oxindole-3-acetyl-amino acid conjugates differ in various plant species
Source: Front Plant Sci. 2023 Jul 18;14:1217421. doi: 10.3389/fpls.2023.1217421 (PMC10390838; doi:10.3389/fpls.2023.1217421)
Supplement: Supplementary file 6 [file Table_5.pdf]

Supplementary Table 5: IAA metabolite profiles in Arabidopsis, maize, wheat, and pea.
Free IAA, oxIAA and their low-molecular-weight conjugates with amino acids and glucose were determined (pmol/g FW) in the four plant models at development stage 1.0. The levels of IAA and oxIAA conjugates with individual amino acids (Asp, Glu, Leu and Phe) were summed up into two corresponding groups IAA-AA and oxIAA-AA, respectively. The distribution (%) of different conjugate classes was calculated as their relative abundance to the total measured IAA metabolite pool (%). All samples were measured in five biological replicates. <LOD, under the limit of detection.

		**IAA**		**IAA-AA**		**IAA-glc**		**oxIAA**		**oxIAA-AA**		**oxIAA-glc**	
		(pmol/g)	(%)	(pmol/g)	(%)	(pmol/g)	(%)	(pmol/g)	(%)	(pmol/g)	(%)	(pmol/g)	(%)
Arabidopsis	shoot	104.4	0.86	69.5	0.6	157.9	1.3	1 863.0	15.3	97.7	0.8	9 869.6	81.2
	root	58.8	1.65	40.0	1.1	94.7	2.7	835.7	23.5	102.9	2.9	2 421.9	68.1
Maize	shoot	16.7	14.19	1.5	1.3	<LOD	-	93.0	78.8	6.8	5.7	<LOD	-
	cotyledon	2 638.2	24.42	76.2	0.7	<LOD	-	7 006.8	64.8	197.9	1.8	885.8	8.2
	root	159.5	49.64	16.6	5.2	<LOD	-	111.0	34.5	34.2	10.6	<LOD	-
Wheat	shoot	18.3	10.33	2.4	1.3	<LOD	-	156.7	88.3	<LOD	-	<LOD	-
	cotyledon	70.0	40.00	43.9	25.1	<LOD	-	61.2	34.9	<LOD	-	<LOD	-
	root	73.9	11.56	127.5	19.9	<LOD	-	220.1	34.4	31.7	5.0	186.1	29.1
Pea	shoot	84.3	10.04	181.3	21.6	53.5	6.4	71.9	8.6	448.6	53.4	<LOD	-
	cotyledon	14.5	0.005	818.8	0.3	<LOD	-	1 412.3	0.5	297 723.7	99.3	<LOD	-
	root	900.0	19.55	667.1	14.5	69.1	1.5	282.4	6.1	2 683.8	58.3	<LOD	-
